# An Explorative Study to Assess the Suicidal Risk Amongst Infertile Patients

**DOI:** 10.1192/bjo.2022.193

**Published:** 2022-06-20

**Authors:** Baidyanath Ghosh Dastidar

**Affiliations:** Calcutta National Medical College, Kolkata, India

## Abstract

**Aims:**

To assess the psychosocial impact of infertility amongst female infertile patients including suicidal risk/ suicidal ideation in the given study population.

**Methods:**

A total of 300 women attending the Obstetrics and Gynecology out patients department of a tertiary hospital in Kolkata, India were selected by simple random sampling. 100 fertile women attending the routine ante natal clinic were selected as cases and 100 infertile women seeking fertility treatment were selected as controls. 100 women didn't follow up with the study. The following questionnaires were administered to both case and control group- BAI, BDI, SCL-90-R, SF-36, MINI and socio demographic proforma ; by trained clinic psychologist .

The raw scores & adjusted scores were analysed statistically by SPSS using the following tests, independent t test, chi square test and Z test.

**Results:**

The results of the MINI scale indicate that up to 25% of the infertile cohort suffer from suicidal risk/ suicidal ideation which is statistically significant in comparison to the control group.

The other psychosocial parameters are also statistically significant in the case in comparison to the control population.

**Conclusion:**

Although the psychosocial impact of infertility has been well researched and documented. Few studies have been conducted globally which assess suicidal risk amongst infertile patients.

Our results corroborate earlier studies such as the Danish administrative population-based registry study by Trille Kristina Kjaer et al which found a causative link between infertility and suicidal risk.

Further research is needed in this direction.

